# Diffuse Pericardial and Diaphragmatic Involvement of Alveolar Echinococcosis in a Liver-Transplanted Patient

**DOI:** 10.1590/0037-8682-0480-2025

**Published:** 2026-02-06

**Authors:** Yener Aydin, Elif Gozgec, Ugur Kaya

**Affiliations:** 1 Ataturk University, Medical Faculty, Department of Thoracic Surgery, Erzurum, Turkey.; 2 Ataturk University, Medical Faculty, Department of Radiology, Erzurum, Turkey.; 3 Ataturk University, Medical Faculty, Department of Cardiovascular Surgery, Erzurum, Turkey.

**FIGURE 1:**
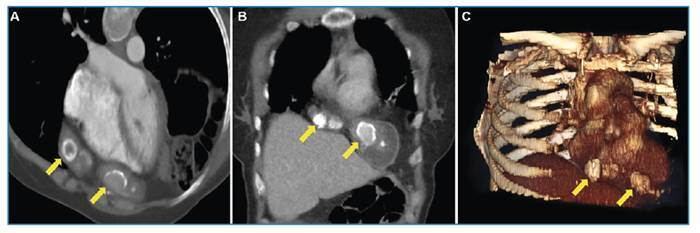
Contrast-enhanced thorax CT images obtained in the axial oblique **(A)** and coronal **(B)** planes, as well as 3D oblique anterior-posterior reconstruction images **(C)**, showing a central hyperdense lesion (arrows) in the pericardial area extending to the diaphragm.

A 40-year-old woman presented to our hospital with chest pain. She had undergone liver transplantation 5 years ago for alveolar echinococcosis. Radiological imaging revealed a hyperdense lesion in the pericardial area, characterized by dense calcified areas extending to the diaphragm (Figure 1). The patient underwent median laparotomy, and the lesion was resected en bloc with portions of the diaphragm and pericardium. The pericardial defect was reconstructed using the bovine pericardium, and the diaphragmatic defect was repaired using a Prolene mesh (Ethicon; Somerville, NJ, USA). Alveolar echinococcosis was confirmed histopathologically by identifying laminated membranes using periodic acid-Schiff (PAS) staining. The postoperative course was uneventful, and the patient was discharged on postoperative day 7. 

Alveolar echinococcosis is a life-threatening parasitic disease caused by *Echinococcus multilocularis*, which primarily affects the liver. Early stage management relies on radical surgical resection combined with adjuvant chemotherapy to achieve the best outcomes; however, liver transplantation remains the only curative treatment for advanced alveolar echinococcosis^1^ ^,^ ^2^. Albendazole, the most widely used benzimidazole agent, is administered to prevent recurrence in surgically managed cases and to suppress parasitic growth in patients in whom surgery is not feasible^1^. In rare cases, recurrence of alveolar echinococcosis with calcified lesions can occur after transplantation, potentially involving extrahepatic sites^3^. Surgical resection should be considered as an effective approach for managing pleural and pericardial recurrences of alveolar echinococcosis after liver transplantation.
